# Computational designing of a peptide that potentially blocks the entry of SARS-CoV, SARS-CoV-2 and MERS-CoV

**DOI:** 10.1371/journal.pone.0251913

**Published:** 2021-05-18

**Authors:** Priya V. K., Satish Prasad Rath, Parvin Abraham

**Affiliations:** 1 National Institute of Technology, Calicut, Kerala, India; 2 MIMS Research Foundation, Calicut, Kerala, India; Kingston University, UNITED KINGDOM

## Abstract

Last decade has witnessed three major pandemics caused by SARS-CoV, SARS-CoV-2 and MERS-CoV that belong to Coronavirus family. Currently, there are no effective therapies available for corona virus infections. Since the three viruses belong to the same family and share many common features, we can theoretically design a drug that can be effective on all the three of them. In this study, using computational approach, we designed a peptide (Peptide 7) that can bind to the Receptor Binding Domain (RBD) of SARS-CoV, SARS-CoV-2 and MERS-CoV thereby preventing the entry of the viruses into the host cell. The peptide inhibitor was designed as a consensus peptide from three different peptides that might individually bind to the RBD of the three viruses. Docking studies and molecular dynamic simulations using Peptide 7 has shown that it binds with higher affinity than the native receptors of the RBD and forms a stable complex thereby preventing further viral-receptor interaction and inhibiting their cellular entry. This effective binding is observed for the three RBDs, despite the Peptide 7 interactions being slightly different. Hence; this peptide inhibitor can be used as a potential candidate for the development of peptide based anti-viral therapy against Corona viruses.

## Introduction

The COVID-19 pandemic continues to be one of the most dreadful diseases, affecting almost all the countries and challenging the entire social and economic status of the world. It has become a global public health issue now. It is caused by severe acute respiratory syndrome-2 (SARS-CoV-2) virus and exhibits human to human transmission. Coronaviruses belong to the Coronaviridae family and are included in the order of Nidovirales. They are mainly classified into three major genera called α, β, and γ [1, 2]. SARS-CoV, SARS-CoV-2 and MERS-CoV viruses fall into β-coronaviruses genera and considered to be highly pathogenic to human. SARS-CoV caused the SARS epidemic in 2002 to 2003, reporting over 8,000 infections with a fatality rate of ∼10% [3]. In 2012, MERS-CoV emerged from the Middle East region. As of 16 October 2014, MERS-CoV had caused a fatality rate of ∼36%including 877 infections [4, 5]. Coronavirus virions contain an envelope, a helical capsid, and a single-stranded and positive-sense RNA genome. The length of their genomes are the largest among all RNA viruses, typically ranges between 27 and 32 kb [6].

The first and foremost step by which a virus enters a cell is by recognising a specific host cell receptor. In case of SARS-CoV and SARS-CoV-2, an envelope-anchored spike protein (S) mediates cellular entry by first binding to a host ACE-2 receptor and then fusing viral and host membranes. The spike (S) protein can be divided into three segments (i) an ectodomain (ii) a single pass trans membrane anchor and (iii) a short intra cellular domain. The ectodomain is further divided into a receptor binding S1 domain and a membrane fusion S2 domain [7]. S1 domain consists of an N-terminal (S1-NTD) and a C-terminal (S1-CTD), either or both of these regions can act as a receptor binding domain (RBD). The 223 amino acid region of RBD resides within the S1 subunit while the S2 subunit region consist of a proximal fusion peptide (FP), followed by a heptad region 1 and 2 (HR1 and HR2) and a trans membrane domain (TM) and a distal cytoplasmic tail [7, 8]. The fact that, highly similar Coronavirus S1-CTDs within the same genus can recognize different protein receptors, whereas very different coronavirus S1-CTDs from different genera can recognize the same protein receptor makes understanding of their receptor binding studies much complex. For example, though SARS-CoV and MERS-CoV both belong to the same β-genus, MERS-CoV S1-CTD recognizes dipeptidyl peptidase 4 (DPP4) [9] and SARS-CoV and SARS-CoV-2 recognises ACE-2 receptors [10, 11].

Following ACE-2 binding, a substantial structural rearrangement of the S-protein allows the viral membrane to fuse with the host cell membrane [12, 13]. The prefusion trimer—receptor binding causes the shedding of the S1 subunit and the corresponding transformation of S2 subunit into a stable post fusion conformation. For receptor binding to occur, RBD of S1 undergoes a hinge-like conformational movement that results in the transient hide or expose of the region of receptor binding to occupy a receptor. These two different conformational states are denoted as the “down” and the “up” states, where down refers to the receptor-inaccessible state and up refers to the receptor-accessible state, which is thought to be less stable [14–16]. Finally, it is the formation of a six-helix bundle (6-HB) fusion core formed by the interaction of the heptad repeat 1 (HR1) and 2 (HR2) domains in its S2 subunit that brings the viral and cellular membranes into close proximity for facilitating fusion. HR1 domain exhibits 92.6% identity with SARS-CoV with an eight amino acid residue difference and 100% overall identity with HR2 [17].

Remdesivir is the only anti-viral FDA approved drug for COVID-19 treatment. Acting as a nucleoside analogue, it inhibits the RNA-dependent RNA polymerase (RdRp) of Coronaviruses including SARS-CoV-2 [18, 19]. The current ongoing vaccines against SARS-CoV-2 are based on mRNA, DNA, subunit and viral vectors [20, 21]. So, there is an urgent need to develop effective anti-viral therapies to combat this issue. Designing structure-based inhibitors that can disrupt or block the specific viral-receptor interaction is one of the effective strategies to block the viral entry. Although several of small molecules have been screened computationally, they are not found to be effective at blocking protein-protein interaction (PPI). Peptide inhibitors, on the other hand, can disrupt PPI effectively by specifically binding to the interface binding region [22, 23]. They are low immunogenic in nature, which makes them a suitable candidate for anti-viral therapy. Recently, Zhang *et al*. [24] reported a 23-mer peptide extracted from ACE-2 α1 helix region, that binds SARS-CoV-2 RBD with low nanomolar affinity. Similarly, Han *et al*. [25] designed certain inhibitor peptides from the protease domain of ACE2.

In this study, we have designed a peptide inhibitor that can bind the RBD of SARS-CoV, SARS-COV-2 and MERS irrespective of the differences in the type of receptors they bind. This interaction between the spike protein RBD and the peptide thus help to prevent the binding of S protein to the respective receptor, inhibiting the entry of these viruses. Using computational methods, we were able to show that the peptide-protein complex is stable and shows a higher binding affinity as compared to the protein-receptor complex. This study may pave a way for the development of peptide-based drugs in the treatment of Coronavirus infections.

## Materials and methods

### Modeling the structures

The crystal structures for the Receptor Binding Domain in complex with its receptors for SARS-CoV-2 (6M0J [7] and 6LZG [26], SARS- CoV (2AFJ [27]) and MERS-CoV (4L72 [28]) were downloaded from the Protein Data Bank. The structure 2AJF has missing residues which were modeled using SWISS-MODEL [29]. 6LZG, 2AJF and 4L72 were submitted to Rosetta Peptiderive server [30] to obtain 10 residue long peptide inhibitors. The 10 residue length is the default value for the server and we used it since most peptide drugs are 10 residues or less [31], and also we were deriving a consensus sequence and did not want a lengthy consensus peptide. A 16 residue long consensus pattern was derived from the three linear peptides that were derived from the three structures respectively, using JalView [32] and manual assignment. We generated a list of 32 peptides that satisfied the generated pattern. The peptides obtained from Peptiderive and the consensus pattern is listed in Table 1 and the 32 peptides are listed in S1 Table.

**Table 1:** Potential peptide sequences that may bind to the RBDs.

Name	Peptide Sequence
SARS-CoV-2	KTFLDKFNHE
SARS-CoV	LGKGDFRILM
MERS-CoV	APASMLIGDH
Consensus sequence	APA(S/K)(M/T)(L/F)LGK(F/G)DH(E/R)ILM

### Docking

Docking of these peptides were carried out against the RBD in three structures, 6M0J, 2AJF and 4L72 using HPEPDOCK [33], using the binding sites derived from literature [7, 28] and PISA [34]. The best docked peptides for SARS-CoV2 were first selected by calculating the binding energy (ΔG) and dissociation constants (k_d_) using the PRODIGY [35] server for the top 10 poses. The peptides and binding poses with the lowest binding energy as compared to the RBD-ACE2 complex were selected for SARS-CoV2 RBD and for the same peptide their binding energy for SARS-CoV and MERS-CoV RBDs were found. The peptides and poses with lower binding energy as compared to their RBD-receptor complex for all the three RBDs were selected. These selected RBD-peptide structures were further refined with GalaxyRefineComplex [36] and energy minimization was done using Chimera [37] and their binding energies were again calculated using PRODIGY server. These three refined structure were used for further analysis. The residues interacting between the RBD and peptides were found using PPCheck [38] and PISA.

### MD simulations

GROMACS 2018.1 [39] was used for the molecular dynamic simulation of the protein-peptide docked complex. The docked structure was placed in a box which extends 1 nm in all the directions. Charmm36m force field was used and TIP3P water model was used to model water. Na and Cl ions were used to counter the charges in the box. Ensemble equilibration was carried out at NVT at 300K and NPT at 1 bar. Ewald method was used to model the long-range electrostatic interactions. Constant pressure simulations were done using the Parrinello-Rahman method. The simulations were run for 80 ns with dt at 2 fs [40, 41].

## Results and discussion

### Prediction of the peptides

**Fig 1:**
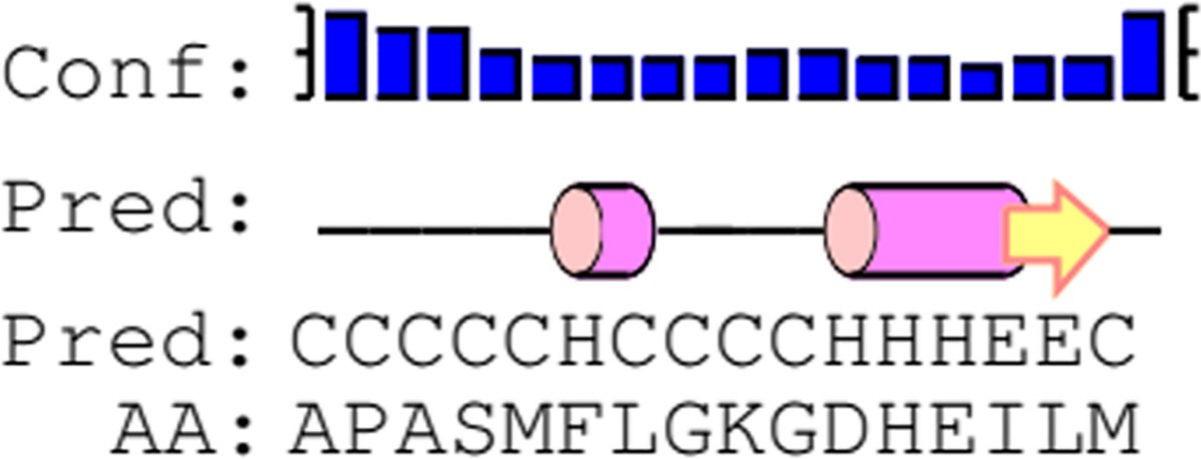
Secondary structure of Peptide 7.

The crystal structures for SARS-CoV-2 (6M0J and 6LZG), SARS- CoV (2AFJ) and MERS-CoV (4L72) were submitted to Rosetta Peptiderive Server. The peptides that can bind to the individual RBDs based on their sequence and their three dimensional structures were predicted (Table 1). A consensus sequence pattern was derived from these three sequences to obtain the final peptide. From the consensus sequence pattern, 32 sequences were derived (S1 Table). All the sequences were docked to the RBDs of 6M0J, 2AJF and 4L72 using the local docking method in HPEPDOCK. The top ten poses from the docked structures were selected for each RBDs and their binding energy was calculated using the Prodigy server and compared with the binding energy of the viral RBD—native receptors. The peptides with binding energy near or lower than that of their native receptors were selected for further analysis. The selected protein-peptide structures were further refined by using GalaxyRefineComplex and energy minimization was carried out using Chimera. There were multiple peptides that had a lower binding energy than the native receptors, but out of these, only two peptides (6 and 7) were found to be favorable for all three RBDs, and out of these two, Peptide 7 was taken for further analysis. The binding energy of the refined docked structure of Peptide 7-RBD complex and the native receptor-RBD are given in Table 2. The secondary structure of the Peptide 7 was predicted using Pep2D [42] (Fig 1). It consists of coil and did not show any defined secondary structure.

**Table 2:** Comparison of the binding energy (ΔG) between the native receptor and Peptide 7.

Sequence	SARS-CoV-2	SARS-CoV	MERS-CoV
Native receptor	-11.9	-10.8	-9.9
Peptide 7: APASMFLGKGDHEILM	-12.2	-11.2	-14.6

All values are in kcal mol^-^1.

### Docking and molecular dynamic simulations

The Peptide 7 is a consensus sequence that can bind to the three different RBDs of SARS-CoV, SARS-CoV-2 and MERS-CoV. The docked structures of the RBDs and Peptide 7 were further analyzed using Molecular dynamic simulations to understand their interaction in the complex.

#### SARS-CoV-2

**Fig 2:**
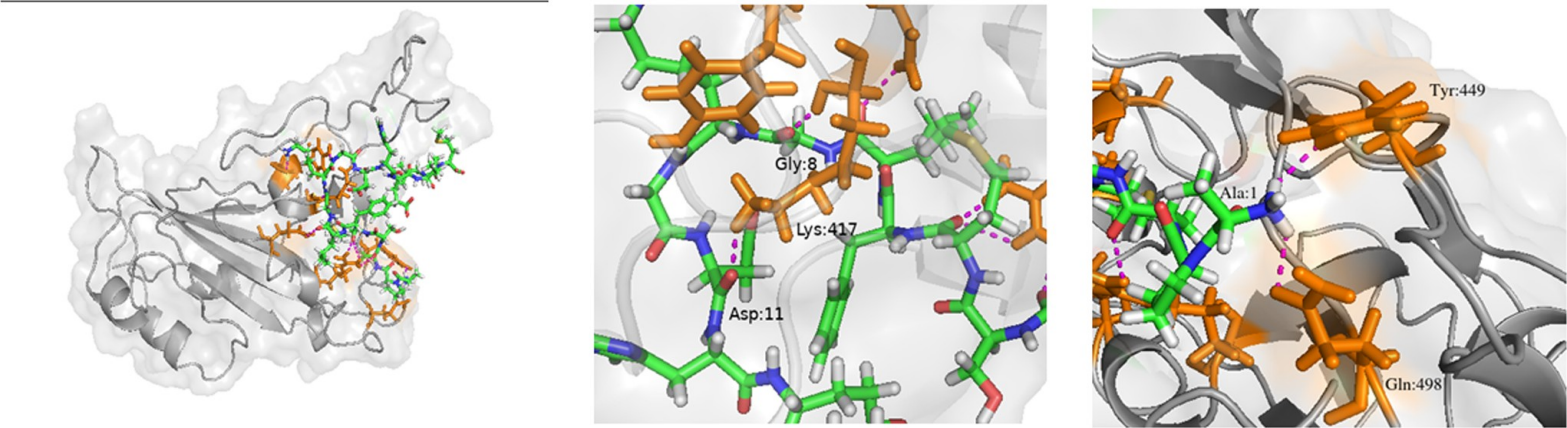
(a) Peptide 7-SARS-CoV-2 RBD complex. Peptide 7 (green, blue and white liquorice), RBD (grey) and residues in RBD which interact with Peptide 7 are highlighted (orange liquorice). (b) Hydrogen bonding between Peptide 7 Gly8 and Asp11 with RBD Lys417. (c) Hydrogen bonding between Ala1 and Tyr449.

The structure of SARS-CoV-2 RBD was taken from 6M0J and Peptide 7 was docked locally onto to the ACE2 receptor binding region of the spike protein using HPEPDOCK. Out of the 10 best poses for the docked structure, the pose with the binding constant lower than that of the native receptor was selected (Fig 2A). This docked structure was further refined using GalaxyRefineComplex and Chimera. This method was followed in case of SARS-CoV and MERS-CoV RBDs as well. Analysis of the interface and interactions between SARS-CoV-2-RBD and Peptide 7 using PPCheck and PISA showed that there are eight hydrogen bonds between the Peptide 7 and residues on the interface of the RBD. The interactions are spread more towards the N-terminal region of the peptide. Peptide 7 residues Ala1 interacts with Tyr449, Ala3 and Met5 interacts with Arg403, Met7 with Gln409, Gly8 and Asp11 with Lys417, Lys9 with Asp420, Gly10 with Tyr421. Lys9 with Asp420 also shows formation of salt bridge between them. Out of these interactions, the interaction towards Lys417 (Fig 2B) and Tyr449 (Fig 2C) are of particular interest because both have been found to be involved in hydrogen bonding with the ACE2 receptors and hence Peptide 7 might disrupt the binding of the ACE2 receptor to the RBD.

**Fig 3:**
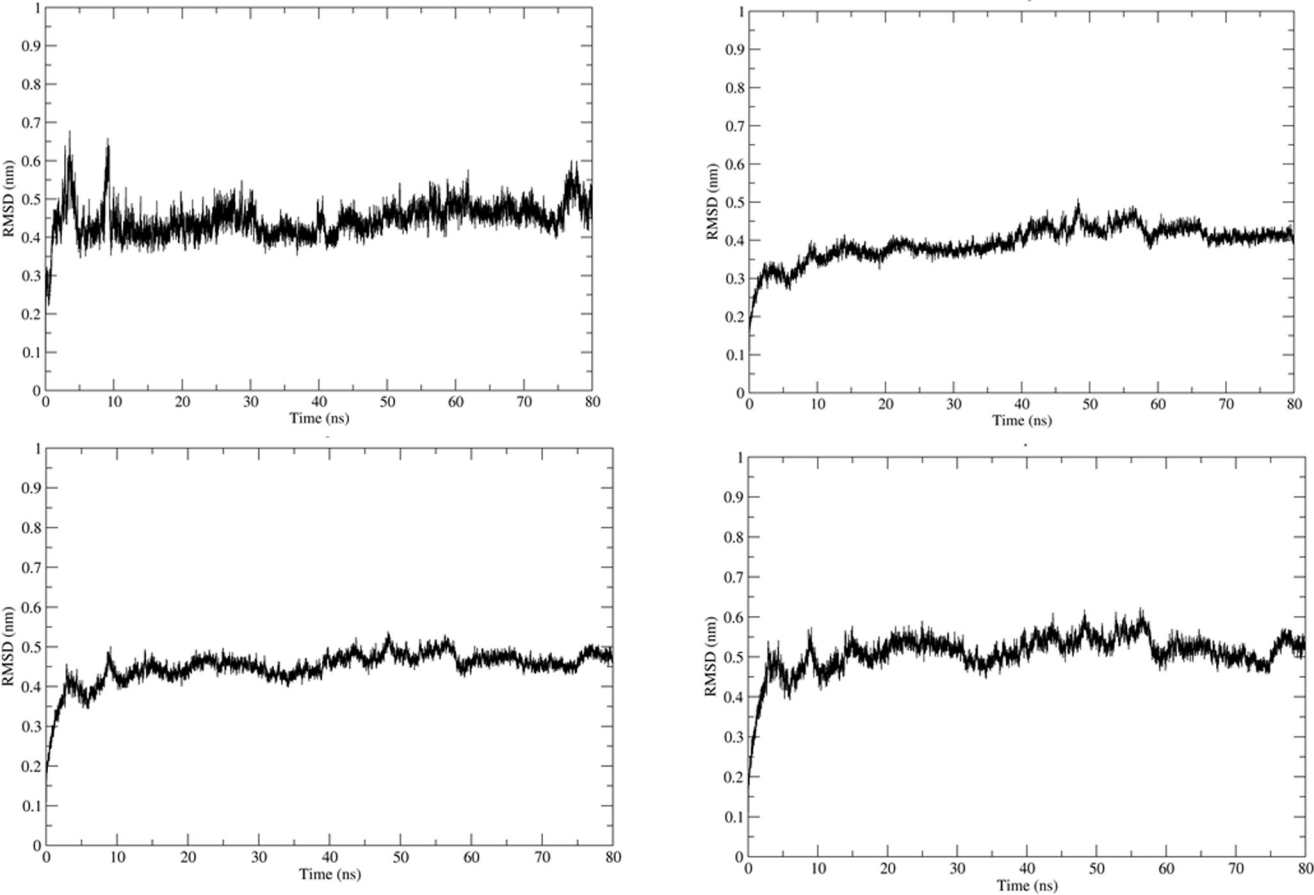
(a) RMSD of Peptide7 in the complex. (b) RMSD plot of SARS-CoV-2 RBD in the complex. (c) RMSD plot of peptide7-SARS CoV-2 RBD (d) Interface of Peptide 7 and SARS-CoV-2 in the complex.

**Fig 4:**
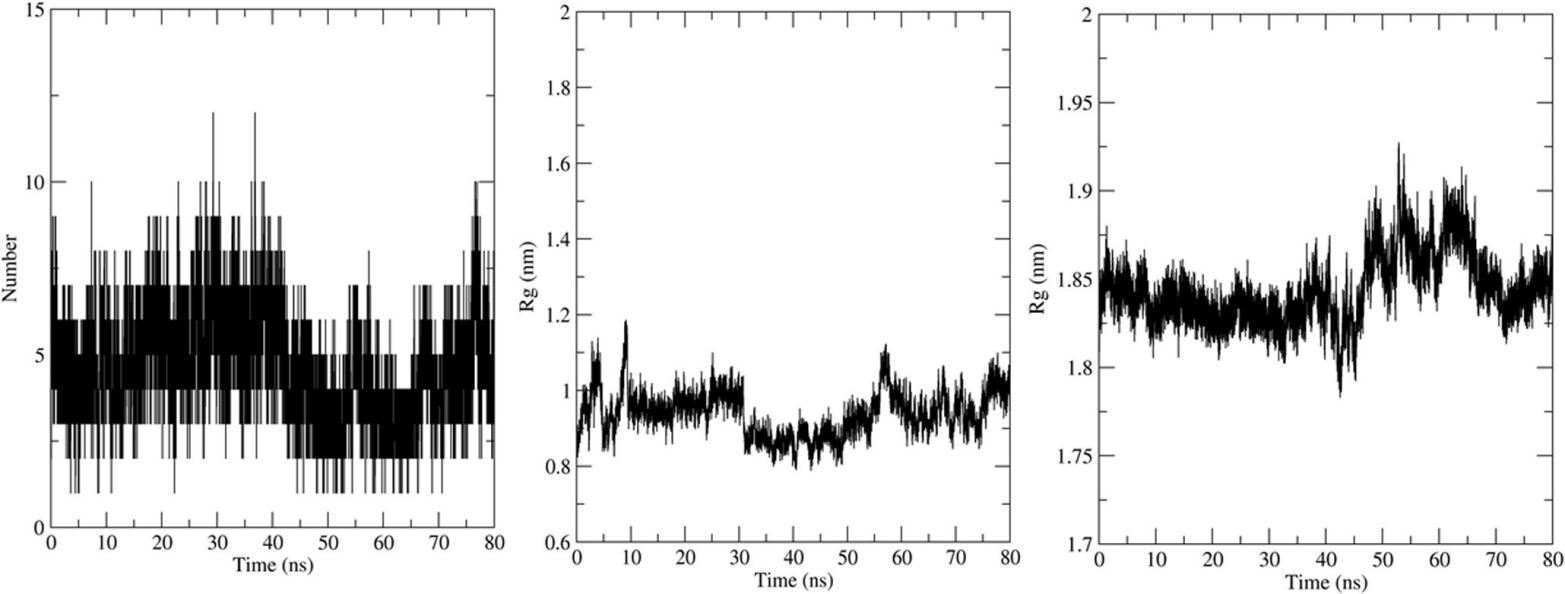
(a) Number of Hydrogen bonds in the MD simulation. (b) Radius of gyration (Rg) of the Peptide 7 in complex and (c) SARS-CoV-2-RBD in complex.

Simulation using GROMACS 2018.1 was carried out to understand the dynamics of the peptide-RBD complex. One Cl^-^ ion was added to neutralize the charge in the structure and MD simulation was run for 80 ns. The RMSD of the peptide for the last 70 ns of the simulation was 0.45 ± 0.04 nm (Fig 3A); this might be due to the lack of interactions between the Peptide 7 residues 12–16 with the RBD and the lack of secondary structure in the peptide. The RMSD of the protein-peptide complex was 0.46 ± 0.02 nm for the last 70 ns of the simulation showing that the peptide-RBD complex is stable (Fig 3C). The residues present in the interface region (S2 Table) were found using InterProSurf [43] and their RMSD was calculated to be 0.52 ± 0.03 nm, which confirmed the interface is stable (Fig 3D). The average number of hydrogen bonds in the last 70 ns of the simulation was calculated to be 4.68 which show a strong attachment between the peptide and RBD (Fig 4A). The compactness and stability of the docked structure was found using the radius of gyration and the structure was found to be stable throughout the simulation without too much fluctuation (Fig 4B and 4C).

#### SARS-CoV

**Fig 5:**
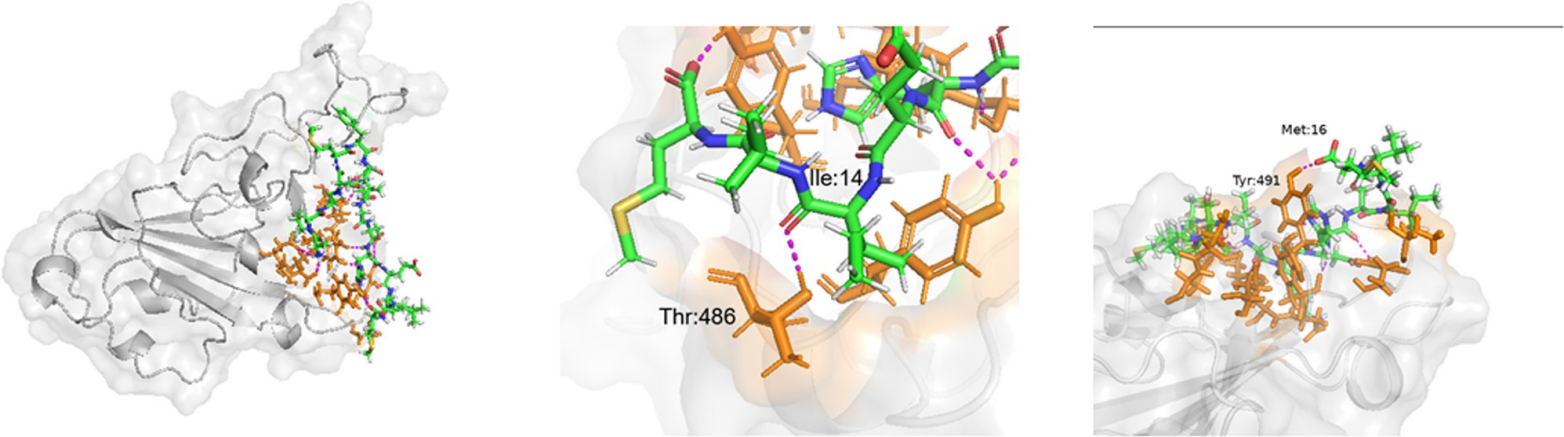
(a) Peptide 7-SARS-CoV RBD complex. Peptide 7 (green, blue and white liquorice), RBD (grey) and residues in RBD which interact with Peptide 7 are highlighted (orange liquorice). (b) Hydrogen bonding between Peptide 7 and RBD residues, Ile14 and Thr486 (c) Hydrogen bonding between Met16 and Tyr491.

The structure of SARS-CoV RBD was taken from 2AJF and the peptide was docked locally onto the ACE2 receptor binding region. The refined structure is as shown in Fig 5A. There are 5 hydrogen bonds according to PISA and PPCheck. The Peptide 7 interacts with SARS-CoV-RBD via; Ala1 with Lys390, Gly8 with Asn479, Lys9 with Asp480, Asp11 with Tyr436 and His12 with Tyr481. Salt bridges are formed between Ala1 with Asp392 and Asp393, Lys9 with Tyr481. It is interesting to note that Thr486 and Tyr491 (Fig 5B and 5C) both interact with the ACE2 receptor and this peptide may be able to block the interaction between them. The interactions between Peptide 7 and RBD are mostly concentrated towards the middle of the peptide.

**Fig 6:**
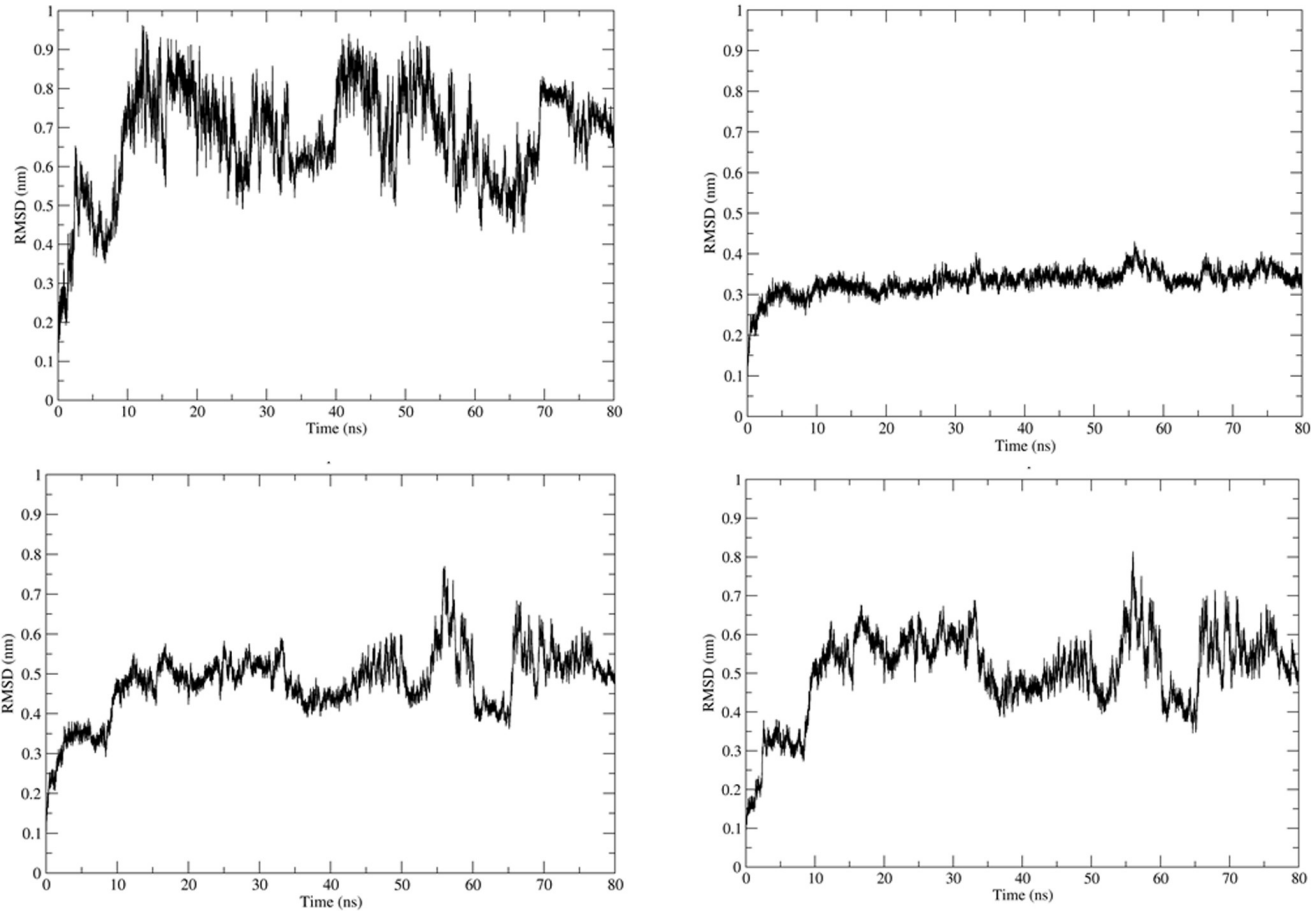
(a) RMSD of Peptide 7 in the complex. (b) RMSD plot of SARS-CoV RBD in the complex. (c) RMSD plot of peptide 7-SARS CoV RBD (d) Interface of Peptide 7 and SARS-CoV in the complex.

**Fig 7:**
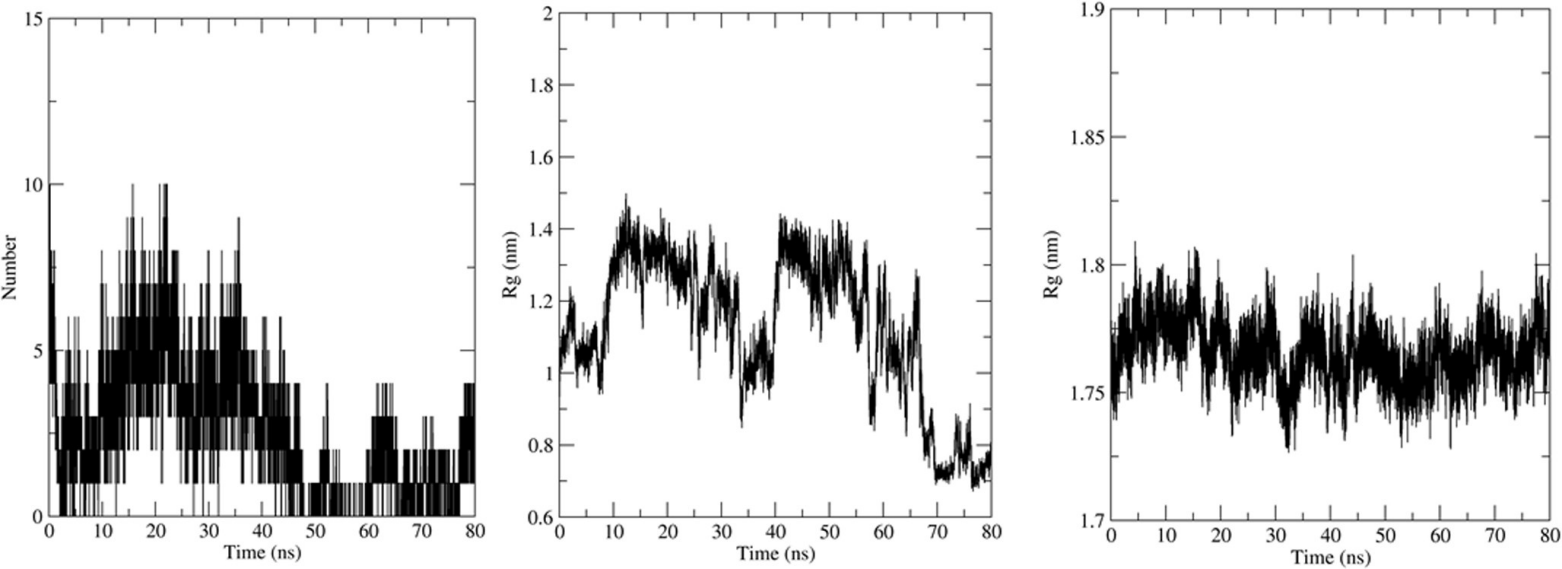
(a) Number of Hydrogen bonds in the MD simulation. (b) The Radius of Gyration of the components of Peptide 7 in the complex and (c) SARS-CoV in the complex.

The molecular dynamics simulation was carried out on the docked structure of SARS-CoV RBD and Peptide 7. One Cl^-^ ion was added to neutralize the charge. The MD analysis was run for 80 ns and several parameters were calculated. The Peptide 7 seems to be stable during the simulation based on the RMSD (Fig 6A) which is on an average 0.7 ± 0.1 nm based on the last 70 ns of the simulation. The RMSD of the peptide7-SARS-CoV is 0.5 ± 0.06 nm showing that the complex is stable (Fig 6C). The RMSD of the SARS-CoV-RBD is 0.34 ± 0.2 nm (Fig 6B) and the interface region is 0.54 ± 0.07 nm (Fig 6D). The number of Hydrogen bonds is 2.54 during the last 70 ns of the simulation showing that there are several hydrogen bonds that hold the peptide and protein together (Fig 7A). The radius of gyration remains stable showing that the complex is compact and stable (Fig 7B and 7C).

#### MERS-CoV

**Fig 8:**
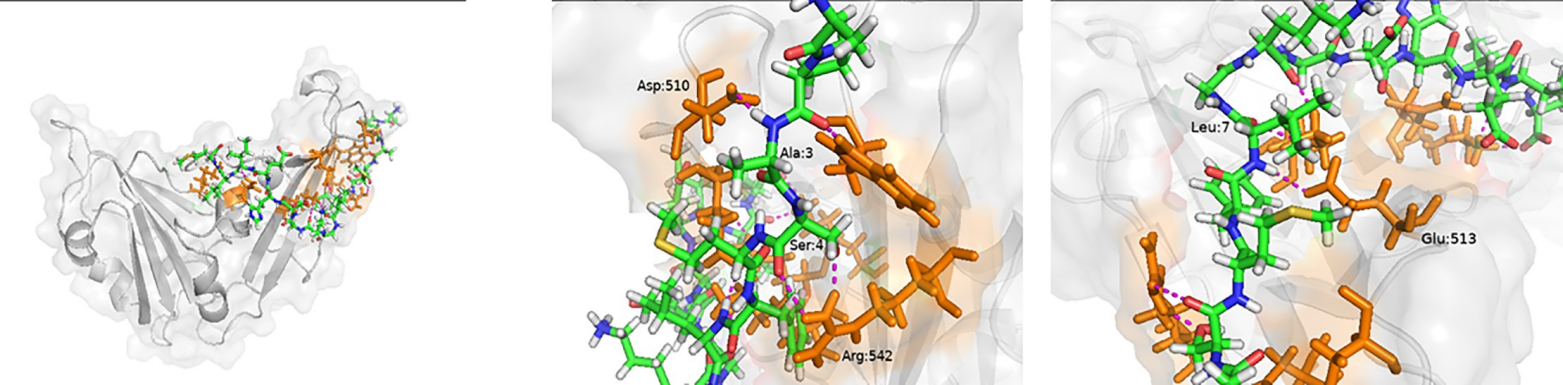
(a) Peptide 7 –MERS-CoV RBD complex. The Peptide 7 residues (blue, green and white). The MERS-CoV RBD is shown in grey and the residues interacting with Peptide 7 are shown in orange. (b) Interaction between MERS-CoV RBD Asp:510 and Peptide 7 Ala:3 and MERS-CoV RBD Arg:542 and Peptide 7 Ser:4. (c) Interaction between MERS-CoV RBD Glu:513 and Peptide 7 Leu:7.

The structure of MERS-CoV RBD was taken from 4L72 and the peptide was docked locally onto the Dipeptidyl peptidase 4 (DPP4) binding region. The refined structure has seven hydrogen bonds between Ala3 with Asp510, Ser4 with Arg542, Leu7 and Lys9 with Lys502, Leu7 with Glu513, Ile14 with Gln466, Leu15 and Met16 with Lys470 (Fig 8A). Salt bridges were observed between Met16 and Lys470. It is interesting to note that Asp510, Arg542 and Glu513 interact with the DPP4 and facilitate in its binding (Fig 8B & 8C) and hence Peptide 7 can possibly prevent it. Here, the hydrogen bonding between Peptide 7 and RBD extend almost the length of Peptide 7 and this includes the C-terminal region also.

**Fig 9:**
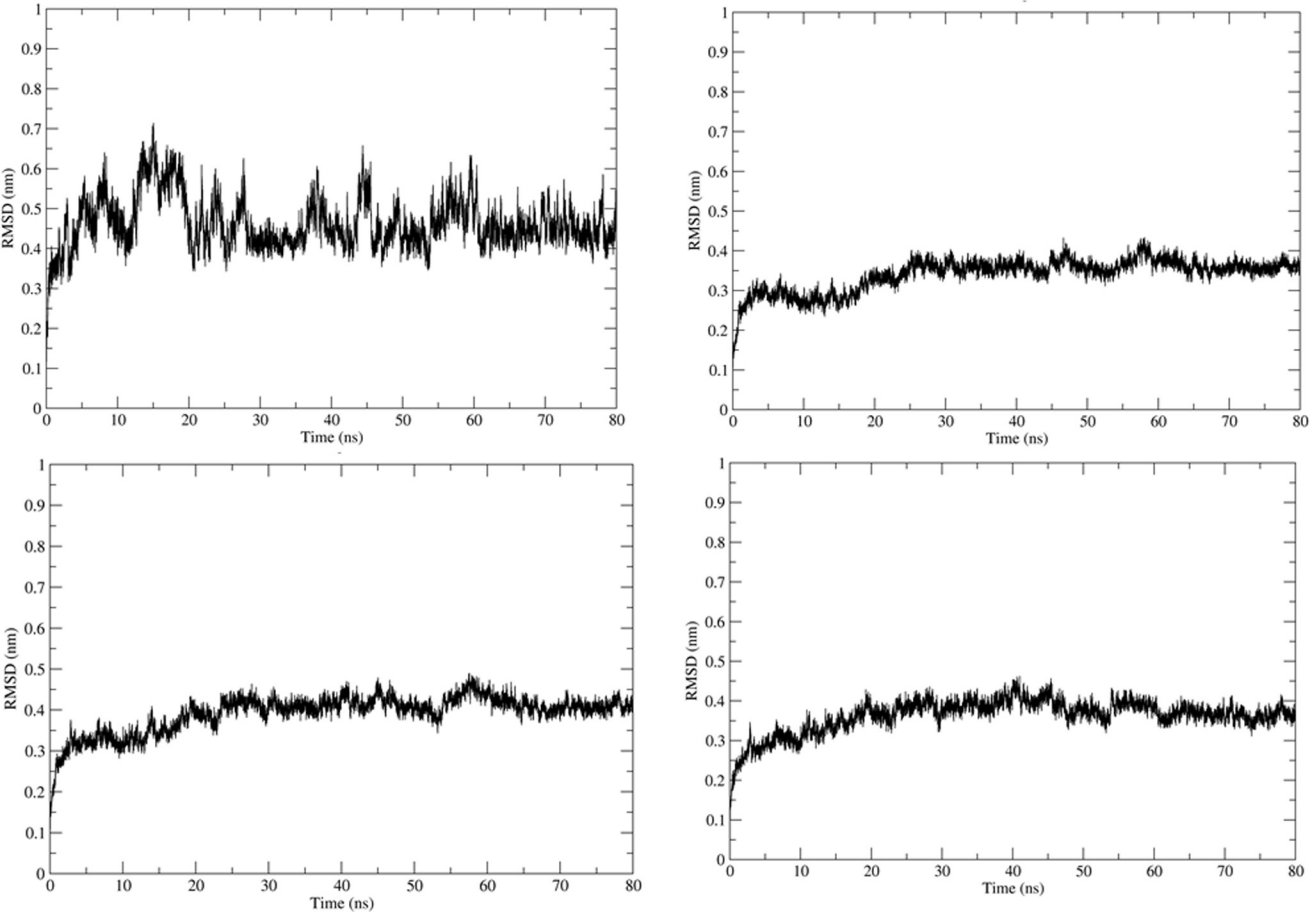
(a) RMSD of Peptide 7 in the complex. (b) RMSD plot of MERS-CoV RBD in the complex. (c) RMSD plot of Peptide7-MERS-CoV RBD (d) Interface of Peptide 7 and MERS-CoV in the complex.

**Fig 10:**
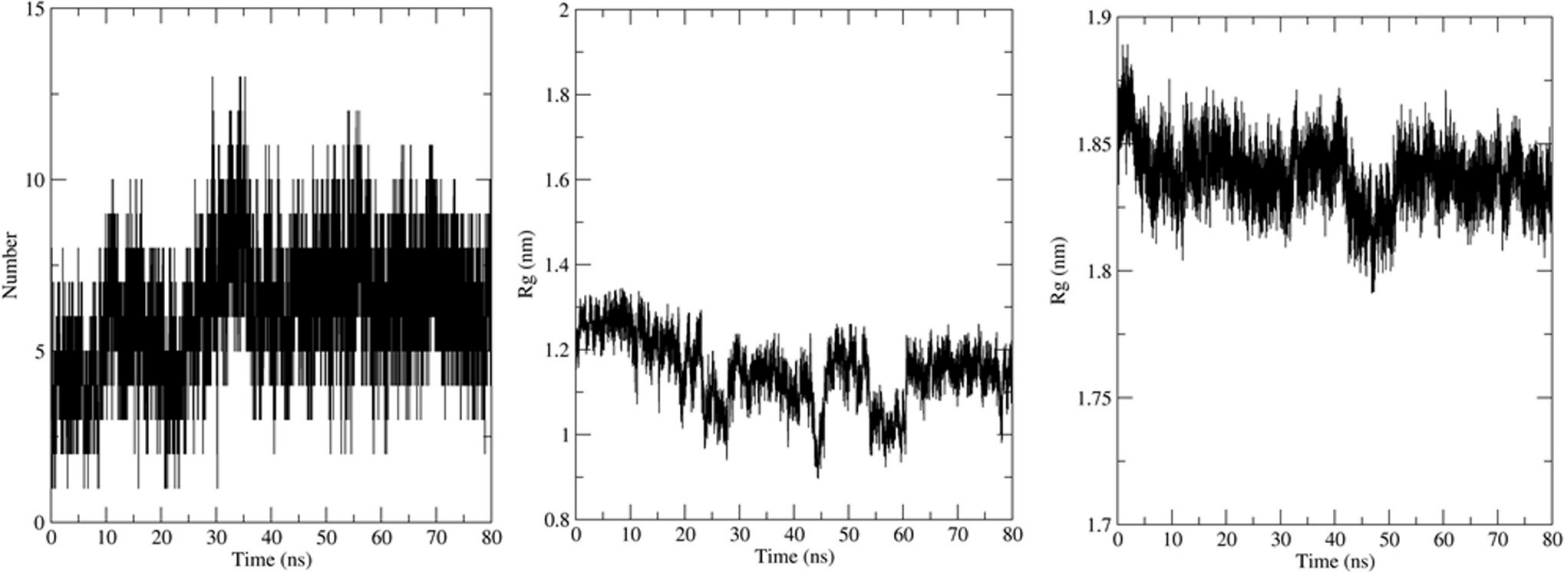
(a) Number of Hydrogen bonds in the MD simulation. (b) Radius of gyration of Peptide 7 in the complex (c) MERS- CoV RBD in the complex.

The MD simulation was carried out to understand the dynamic behaviour of the complex. Here, 3 Na^-^ ion was added to neutralize the charge. The MD analysis ran for 80 ns and various parameters were calculated. The Peptide 7 in the complex seems to be stable throughout the simulation based on the RMSD (Fig 9A) which on an average is 0.47 ± 0.06 nm based on the last 70 ns of the simulation. The RMSD of the peptide7-SARS-COV is 0.04 ± 0.03 nm showing that complex is stable (Fig 9C). The RMSD of MERS-CoV RBD is 0.35 ± 0.03 nm (Fig 9B) and that of the interface region is 0.37 ± 0.03 nm (Fig 9D). The average number of Hydrogen bonds is 6.56 (Fig 10A). The radius of gyration is stable showing that the complex is very stable (Fig 10B and 10C).

**Fig 11:**
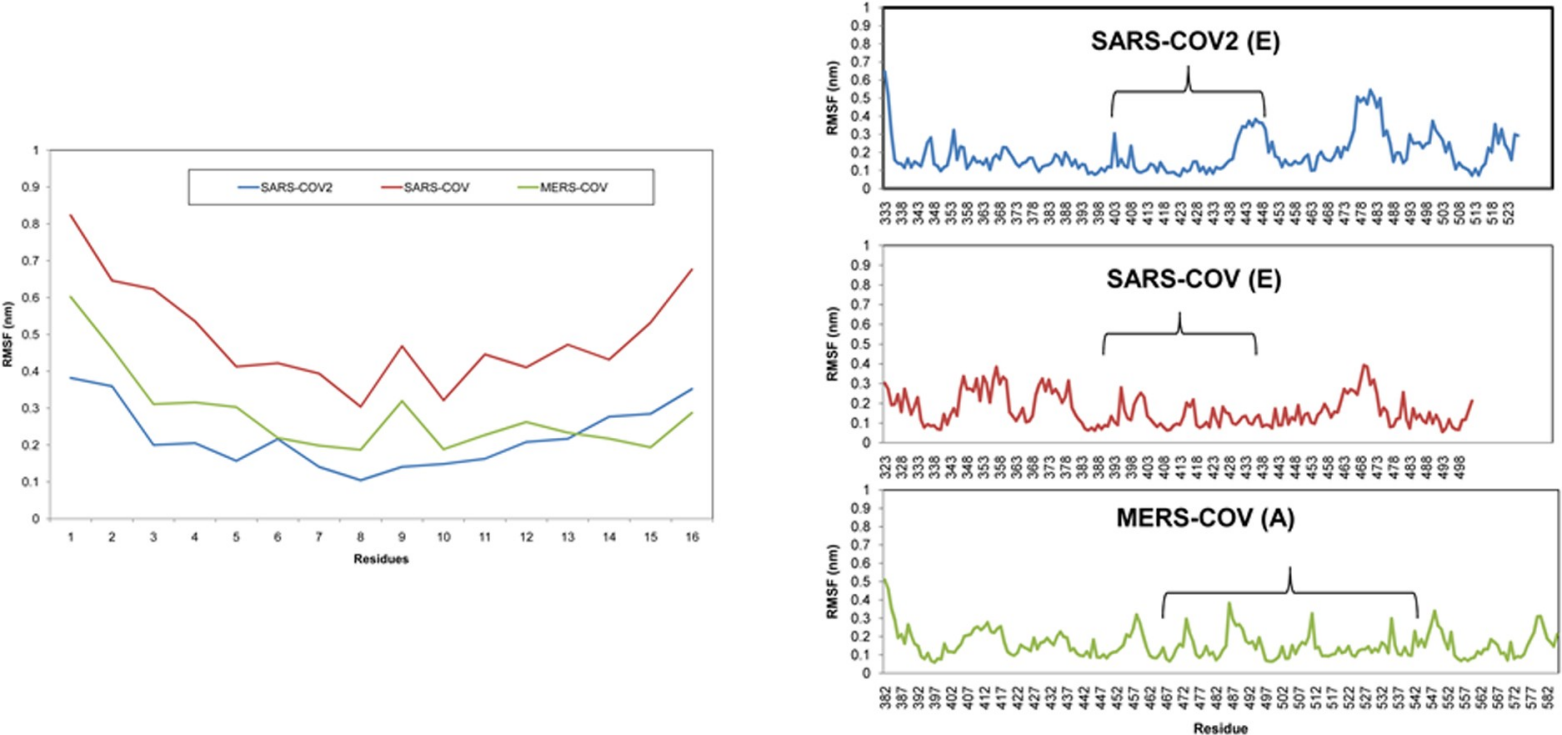
(a) RMSF of Peptide 7 in the Peptide 7- RBD complex. (b) RMSF of the RBD in the Peptide 7-RBD complexes. Brackets show the regions where Peptide 7 binds to the RBD.

Peptide 7 sequence was formed by creating a consensus sequence from three different peptides that bind individually to the three different RBDs and hence the interactions between the Peptide 7 and RBDs differ between them not only due to the difference in the peptides used to create it, but also due to the difference in the target. Even with these differences, the root-mean-square fluctuation (RMSF) of the peptides shows some similarities. While the RMSF is high for Peptide 7 bound to SARS-COV because both its ends do not interact with the RBD, still its RMSF profile is similar to Peptide 7 bound to MERS-COV. The RMSF of Peptide 7 bound to SARS-COV2 and MERS-COV is low because of a large number of interactions that bind them to their respective RBDs even though the RMSF profiles are different for the regions that bind Peptide 7 (Fig 11A & 11B).

**Fig 12:**
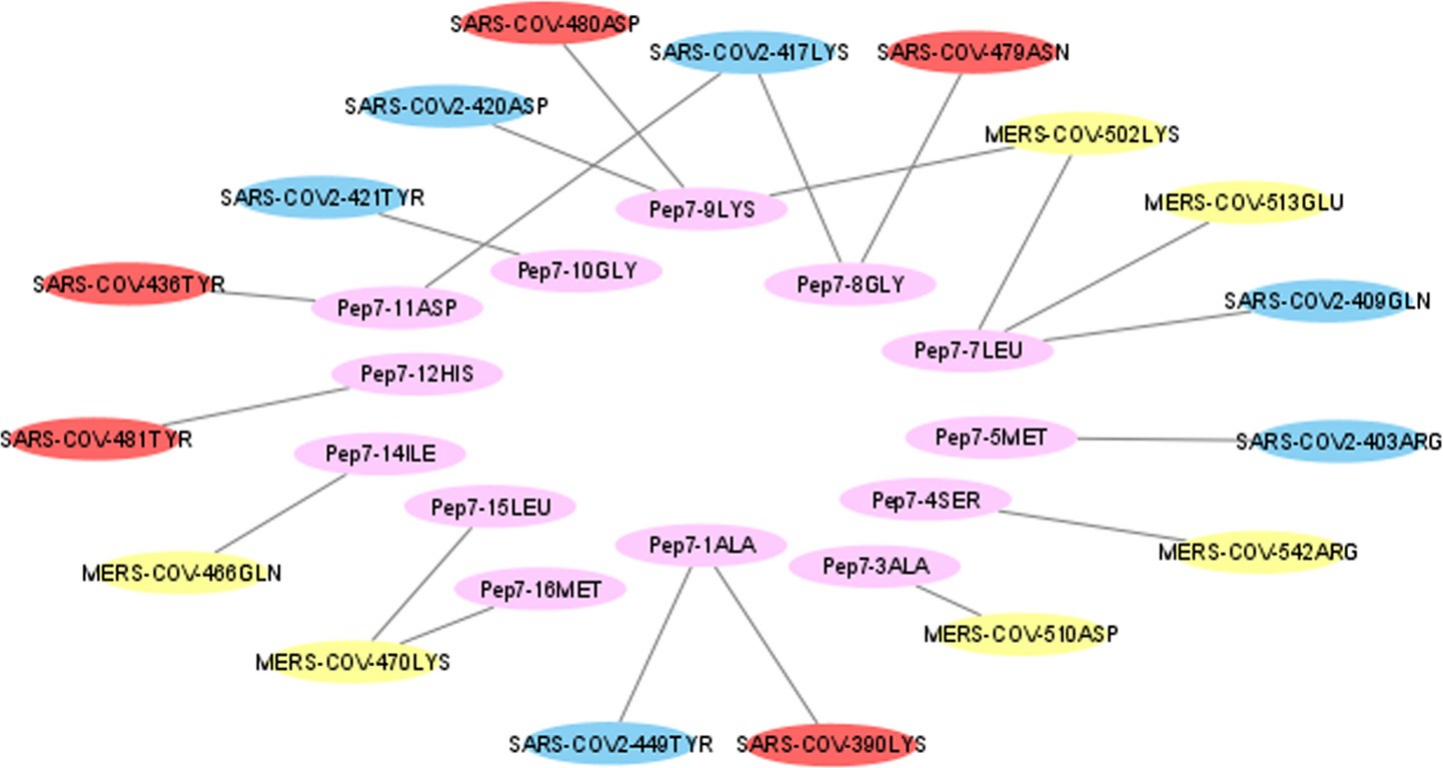
Hydrogen bonds between RBDs and the Peptide 7.

A comparison between the binding of the RBDs and Peptide 7 shows that each of the RBDs binds to distinct residues that other RBDs do not bind. But there are few Peptide 7 residues that interact with more than one RBD; such as Ala1, Leu7, Gly8, Lys9 and Asp11 (Fig 12). This shows that Peptide 7 along with its random coil structure helps in moulding the peptide sequence to the binding region and can possibly inhibit the binding of the native receptors with the RBDs.

## Conclusion

The impact of the ongoing COVID-19 pandemic which started at the end of 2019 will last for many years. SARS-CoV-2, the causative agent of COVID-19 is the seventh member of the *Coronaviridae* family known to infect humans. SARS-CoV and MERS-CoV, two other members of this family, are the causative agents of recent outbreaks of SARS and MERS. As of now, SARS, COVID-19 and MERS do not have any treatment other than supportive care.

In this study, using *in-silico* analysis, we were able to design a 16-mer peptide (Peptide 7) that can bind to the RBD region of SARS-CoV, SARS CoV-2 and MERS-CoV. The sequence was derived by taking a consensus sequence from three distinct peptides that act on the RBD of the three viruses. It has been shown to bind with RBD with higher affinity compared to the native receptor and forms stable complex with the RBD.This peptide was able to interact with the key residues that are involved in binding of the RBDs to their native receptors and thus would be able to prevent the binding and initiation or spread of infection.

Residues like Lys417 and Tyr449 in SARS-CoV2, Thr486 and Tyr491 in SARS-CoV and Asp510, Arg542 and Glu513 in MERS-CoV RBDs are involved in the interactions of the RBD to their respective receptors. Peptide 7 interacts with these residues and prevents the binding of the native receptor. Though the RMSD is high for the peptide, we attribute it to the absence of any secondary structure and flexibility of the molecule that makes it a good candidate to act on the slightly different RBDs. The Radius of gyration and the hydrogen bonds formed show that the complex of RBD-Peptide 7 is compact and stable during the time of the simulation even though the structural fluctuations are seen where there are limited interactions between the peptide and the RBD. Further *in-vitro* and *in-vivo* tests needs to be performed to understand the efficacy and ADMET properties of Peptide 7.

Peptide based drugs are not new, though few in number, most of them are used in the treatment of diabetes, growth deficiency, cancers and some viral diseases [44, 45]. This peptide could be used as a template and its affinity, stability and bioavailability could be improved by the addition of linker molecules or conjugates such as PEG.

## Supporting information

S1 Table List of peptides that were created using the consensus sequence peptide. Bold peptides were found to be active in all the RBDs in the study.
(DOCX)

S2 Table Interface residues of RBD that interact with Peptide 7 with the predicted hydrogen bonds. (DOCX)
