# Supplementary material for: Computational designing of a peptide that potentially blocks the entry of SARS-CoV, SARS-CoV-2 and MERS-CoV
Source: PLoS One. 2021 May 18;16(5):e0251913. doi: 10.1371/journal.pone.0251913 (PMC8130920; doi:10.1371/journal.pone.0251913)
Supplement: S1 Table — Bold peptides were found to be active in all the RBDs in the study. (DOCX) [file pone.0251913.s001.docx]

**S1 Table:** List of Peptides that were created using the consensus sequences. Peptides in bold were found to be active in all the RBDs in the study.

| **Peptide** | **Sequence** |
| --- | --- |
| Peptide1 | APASMLLGKFDHEILM |
| Peptide2 | APASMLLGKFDHRILM |
| Peptide3 | APASMLLGKGDHEILM |
| Peptide4 | APASMLLGKGDHRILM |
| Peptide5 | APASMFLGKFDHEILM |
| **Peptide6** | **APASMFLGKFDHRILM** |
| **Peptide7** | **APASMFLGKGDHEILM** |
| Peptide8 | APASMFLGKGDHRILM |
| Peptide9 | APASTLLGKFDHEILM |
| Peptide10 | APASTLLGKFDHRILM |
| Peptide11 | APASTLLGKGDHEILM |
| Peptide12 | APASTLLGKGDHRILM |
| Peptide13 | APASTFLGKFDHEILM |
| Peptide14 | APASTFLGKFDHRILM |
| Peptide15 | APASTFLGKGDHEILM |
| Peptide16 | APASTFLGKGDHRILM |
| Peptide17 | APAKMLLGKFDHEILM |
| Peptide18 | APAKMLLGKFDHRILM |
| Peptide19 | APAKMLLGKGDHEILM |
| Peptide20 | APAKMLLGKGDHRILM |
| Peptide21 | APAKMFLGKFDHEILM |
| Peptide22 | APAKMFLGKFDHRILM |
| Peptide23 | APAKMFLGKGDHEILM |
| Peptide24 | APAKMFLGKGDHRILM |
| Peptide25 | APAKTLLGKFDHEILM |
| Peptide26 | APAKTLLGKFDHRILM |
| Peptide27 | APAKTLLGKGDHEILM |
| Peptide28 | APAKTLLGKGDHRILM |
| Peptide29 | APAKTFLGKFDHEILM |
| Peptide30 | APAKTFLGKFDHRILM |
| Peptide31 | APAKTFLGKGDHEILM |
| Peptide32 | APAKTFLGKGDHRILM |
