# Supplementary material for: Computational designing of a peptide that potentially blocks the entry of SARS-CoV, SARS-CoV-2 and MERS-CoV
Source: PLoS One. 2021 May 18;16(5):e0251913. doi: 10.1371/journal.pone.0251913 (PMC8130920; doi:10.1371/journal.pone.0251913)
Supplement: S2 Table — (DOCX) [file pone.0251913.s002.docx]

**S2 Table****:** Interface residues of RBD that interact with Peptide 7 with the hydrogen bonds predicted to be between them.

| **Complex** | **Residues** | **Hydrogen bonds** | | |
| --- | --- | --- | --- | --- |
|  |  | **RBD** | **Peptide 7** | **Distance (Å)** |
| SARS-COV2 | *6M0J:*403, 405, 406, 409, 415, 416, 417, 420, 421, 449, 453, 455, 456, 460, 473, 475, 485, 486, 489, 493, 496, 498, 501, 505  *Peptide 7:*1, 2, 3, 4, 5, 6, 7, 8, 9, 10, 11, 12, 13, 15 | 403Arg  403Arg  409Gln  417Lys  417Lys  420Asp  420Asp  449Tyr | 3Ala  5Met  7Leu  8Gly  11Asp  9Lys  9Lys  1Ala | 2.90  2.71  3.08  2.80  2.90  2.86  2.80  2.13 |
| SARS-COV | *2AJF:*390, 392, 393, 396, 402, 404, 408, 436, 440, 442, 443, 462, 475, 476, 479, 480, 481, 484, 486, 487, 488, 491  *Peptide 7:*1, 2, 3, 5, 6, 8, 9, 10, 11, 12, 14 | 390Lys  479Asn  480Asp  481Tyr  436Tyr | 1Ala  8Gly  9Lys  12His  11Asp | 2.70  3.26  2.66  2.96  3.70 |
| MERS-COV | *4L72:*461, 466, 467, 470, 473, 501, 502, 506, 510, 511, 513, 515, 517, 540, 542, 544, 551, 553, 555  *Peptide 7:*2, 3, 4, 5, 6, 7, 8, 10, 11, 12, 13, 14 | 466Gln  510Asp  513Glu  542Arg  470Lys  470Lys  502Lys  502Lys | 14Ile  3Ala  7Leu  4Ser  15Leu  16Met  7Leu  9Lys | 2.80  2.81  3.24  3.00  2.66  2.78  3.24  3.02 |
